# Antisense-induced exon skipping for duplications in Duchenne muscular dystrophy

**DOI:** 10.1186/1471-2350-8-43

**Published:** 2007-07-05

**Authors:** Annemieke Aartsma-Rus, Anneke AM Janson, Gert-Jan B van Ommen, Judith CT van Deutekom

**Affiliations:** 1 Department of Human Genetics, Leiden University Medical Center, Leiden, the Netherlands; 2 Prosensa B.V., Leiden, the Netherlands

## Abstract

**Background:**

Antisense-mediated exon skipping is currently one of the most promising therapeutic approaches for Duchenne muscular dystrophy (DMD). Using antisense oligonucleotides (AONs) targeting specific exons the DMD reading frame is restored and partially functional dystrophins are produced. Following proof of concept in cultured muscle cells from patients with various deletions and point mutations, we now focus on single and multiple exon duplications. These mutations are in principle ideal targets for this approach since the specific skipping of duplicated exons would generate original, full-length transcripts.

**Methods:**

Cultured muscle cells from DMD patients carrying duplications were transfected with AONs targeting the duplicated exons, and the dystrophin RNA and protein were analyzed.

**Results:**

For two brothers with an exon 44 duplication, skipping was, even at suboptimal transfection conditions, so efficient that both exons 44 were skipped, thus generating, once more, an out-of-frame transcript. In such cases, one may resort to multi-exon skipping to restore the reading frame, as is shown here by inducing skipping of exon 43 and both exons 44. By contrast, in cells from a patient with an exon 45 duplication we were able to induce single exon 45 skipping, which allowed restoration of wild type dystrophin. The correction of a larger duplication (involving exons 52 to 62), by combinations of AONs targeting the outer exons, appeared problematic due to inefficient skipping and mistargeting of original instead of duplicated exons.

**Conclusion:**

The correction of DMD duplications by exon skipping depends on the specific exons targeted. Its options vary from the ideal one, restoring for the first time the true, wild type dystrophin, to requiring more 'classical' skipping strategies, while the correction of multi-exon deletions may need the design of tailored approaches.

## Background

Duchenne and Becker muscular dystrophy (DMD and BMD, respectively) are allelic disorders caused by mutations in the *DMD *gene, which codes for dystrophin [1,2]. Duchenne patients are diagnosed at a young age, suffer from rapid and progressive loss of muscle function and mostly die before the age of 30 due to respiratory or heart failure [3]. By contrast, Becker patients are diagnosed at a later age, often remain ambulant until later in life and generally have near normal life expectancies, although more severe cases are known as well [3]. This discrepancy can be explained by the type of mutation: out-of-frame mutations lead to prematurely truncated, non-functional dystrophin proteins and are associated with the severe DMD phenotype, whereas in-frame mutations give rise to internally deleted, but largely functional proteins found in BMD patients [4]. Over 70% of all DMD and BMD patients carry deletions of one or more exons; small mutations resulting in premature stop codons or disrupted splice sites are found in circa 20% of patients, while duplications of one or more exons make up for around 7% of mutations [5].

During the last few years, promising results have been obtained with antisense-mediated restoration of the reading frame as a therapy for Duchenne patients [6-9]. This strategy aims to correct the reading frame by inducing the skipping of specific exons during pre-mRNA splicing, using antisense oligonucleotides (AONs) that interfere with the splicing of the targeted exons. Thus, a Becker-like dystrophin can be introduced and a severe Duchenne converted into a milder Becker phenotype. We have previously shown the applicability of this therapy in cultured muscle cells derived from patients carrying different deletions and point mutations [6,7,10].

Whereas single exon skipping would in theory offer a therapy for over 50% of DMD patients, we have recently shown the feasibility of the induced co-skipping of two or even multiple exons: by targeting the two outer exons of a stretch of exons, we were able to induce multiexon 45 to 51 skipping both in wild type and patient cell cultures. [7]. This type of multiexon skipping further increases the applicability of the exon skipping therapy to over 70%. The therapeutic possibilities of deploying multiexon skipping to create large deletions associated with mild phenotypes are obvious [7,11]. However, we have recently reported that there are significant technical limitations that depend on the targeted stretch of exons, and the length of the flanking introns [12].

**Figure 1:**
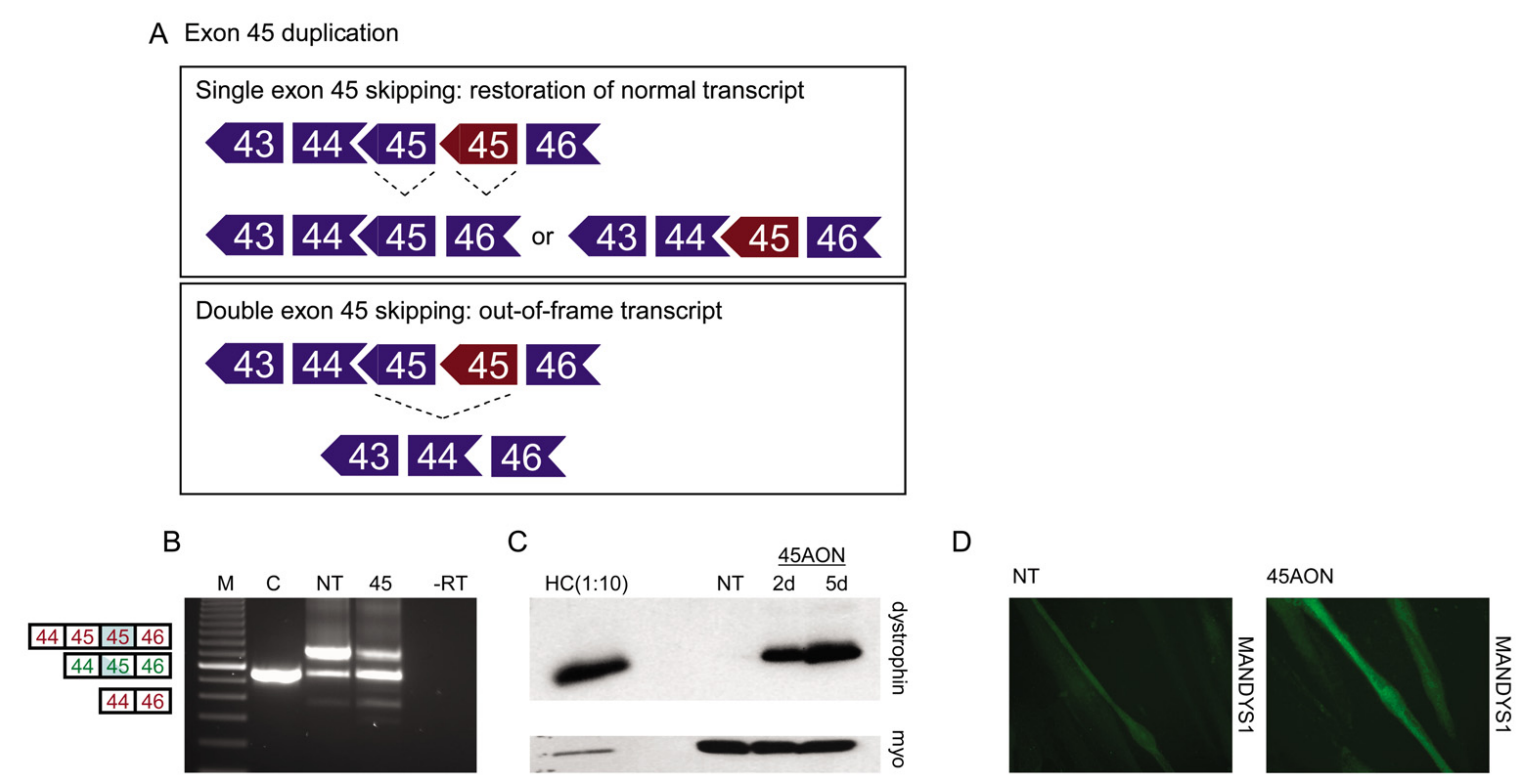
Exon skipping in myotube cultures derived from an exon 45 duplicated patient. A. Schematic overview of the mutation. Original and duplicated exons are shown in blue and red, respectively. Restoration of the wild type transcript can be achieved by skipping of either the original or mutated exon 45 (upper panel) but skipping of both exons 45 results in an out-of-frame transcript (lower panel). B. RT-PCR analysis. Low levels of single exon 45 skipping can be seen in the non treated sample (NT). Exon 45 skipping levels increased significantly after treatment with an exon 45 specific AON (45). Double exon 45 skipping could occasionally be observed at low levels before and after AON treatment. In-frame and out-of-frame transcripts are depicted in green and red, respectively. Duplicated exons are shaded blue. M is 100 bp size standard, C is normal control, -RT is negative control. C. Western blot analysis. Clear dystrophin (Dy4) signals were detected 2 and 5 days (2d and 5d, respectively) after AON treatment, whereas no dystrophin was observed in the non treated sample (NT). The control sample (HC) was diluted 10 times to prevent overexposure. Myosin staining (MF20) was used to confirm equal sample loading (myo). D. Immuno-histochemical analysis. No dystrophin signal was detected in non-treated myosin positive myotubes with either the Dy8 (data not shown) or the MANDYS1 antibodies (NT). After AON treatment (45AON), over 80% of myosin positive cells stained positive for dystrophin (dy8, data not shown, and MANDYS1).

**Figure 3:**
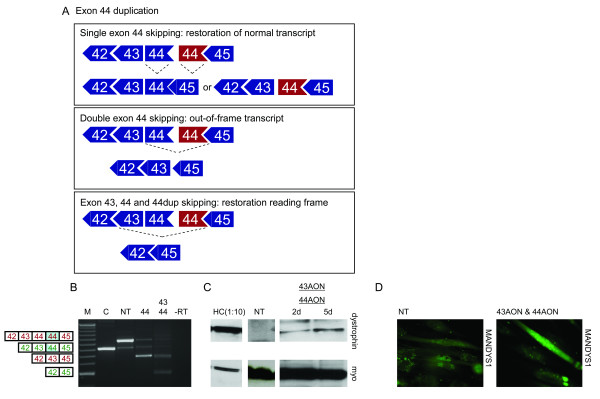
Exon skipping in myotube cultures derived from an exon 44 duplicated patient. A. Schematic overview of the mutation. Original and duplicated exons are shown in blue and red, respectively. Restoration of the normal transcript can be achieved by skipping of either the original or mutated exon 44 (upper panel) but skipping of both exons 44 results in out-of-frame transcripts (middle panel). Skipping exon 43 and both exons 44 will restore the reading frame (lower panel). B. RT-PCR analysis. Low levels of single exon 44 skipping were observed in the non treated sample (NT). After treatment with exon 44 specific AONs (44) both copies of exon 44 were skipped in the majority of the transcripts, generating an out-of-frame product. Using a combination of AONs targeting exon 43 and 44 (43, 44) it was feasible to induce an in-frame transcript where exon 43 is skipped in addition to both copies of exon 44. In-frame and out-of-frame transcripts are depicted in green and red, respectively. Duplicated exons are shaded in blue. M is 100 bp size standard, -RT is negative control. C. Western blot analysis. Dystrophin signals were detected in protein isolated 2 and 5 days (2d and 5d) after AON treatment and not in an untreated sample (NT). The control sample (HC) was diluted 10 times to prevent overexposure. Myosin staining was used to confirm equal sample loading (myo). D. Immuno-histochemical analysis. No dystrophin signal was detected in non-treated myosin-positive myotubes with either the Dy8 (data not shown) or the MANDYS1 antibody. After AON treatment over 70% of myosin positive cells stained positive for dystrophin as detected with Dy8 (data not shown) and MANDYS1.

**Figure 4:**
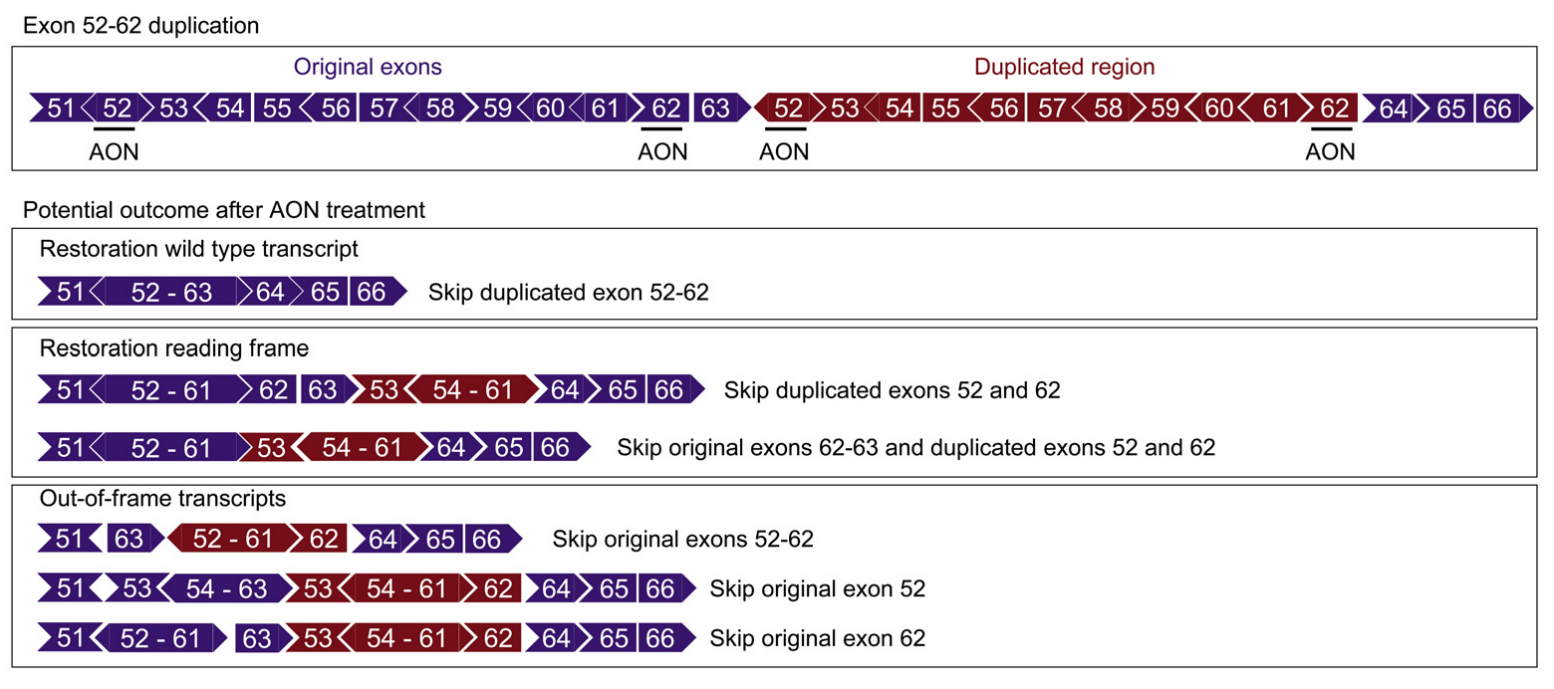
Schematic overview of the exon 52–62 duplication. Original and duplicated exons are shown in blue and red, respectively. The exon 52–62 duplication is located between exons 63 and 64 and therefore only the multiexon skip of the duplicated exons will restore the wild type transcript, whereas multiexon skipping of the original exon 52–62 will result in out-of-frame transcripts (upper and lower panel, respectively). Restoration of the reading frame can be achieved by skipping the duplicated exons 52 and 62, or the original exons 62, 63 combined with the duplicated exons 52 and 62 (middle panel). Note that skipping of the original exon 52 or exon 62 disrupts the open-reading-frame (lower panel).

There is one class of mutations, i.e. the duplication of one or more exons, for which therapeutic antisense-induced exon skipping has not yet been demonstrated. These mutations would actually be ideal targets since the skipping of the duplicated exon(s) would restore normal transcripts allowing the synthesis of full-length, wild type dystrophins (see Figures 1A, 3A and 4). In this study we therefore focussed on antisense-induced exon skipping in cultured cells from patients with two different single exon duplications and from one with a multiple exon duplication.

## Methods

### AONs and primers

AONs (h44AON1, h44AON2, h45AON5, h52AON1 and h62AON1) have been described [13], and consist of 2'-O-methyl RNA and full length phosphorothioate backbones (Eurogentec, Belgium). Primers for RT-PCR were chosen in flanking exons (Eurogentec, Belgium) (see Table 1, sequences upon request).

**Table 1:** Location of RT-PCR primers

Single exon dup^1^	Forward 1	Reverse 1	Forward 2	Reverse 2
Dup exon 44	Exon 41	Exon 47	Exon 42	Exon 45
Dup exon 45	Exon 43	Exon 48	Exon 44	Exon 47

Multiexon dup				

Original exon 52	Exon 50	Exon 54	Exon 51	Exon 53
Duplicated exon 52	Exon 63	Exon 54	Exon 63	Exon 53
Original exon 62	Exon 60	Exon 63	Exon 61	Exon 63
Duplicated exon 62	Exon 60	Exon 66	Exon 61	Exon 65
3' dup breakpoint	Exon 60	Exon 54	Exon 61	Exon 53
Across dup	Exon 63	Exon 66	Exon 63	Exon 65

^1^Dup = duplication

### Myoblast cultures and AON transfection

A primary myoblast culture derived from a DMD patient carrying an exon 45 duplication was provided by Dr. Francesco Muntoni (Imperial college, London, UK) and was cultured as described elsewhere [14]. Myotubes were obtained after 7–14 days of serum deprivation. Fibroblasts (gift from Dr René de Coo, Erasmus Medical Center, Rotterdam, the Netherlands) and amnioblasts (gift from Dr Ieke Ginjaar, Leiden University Medical Center, Leiden, the Netherlands) were available for the patients with an exon 44 duplication and an exon 52–62 duplication, respectively. Following infection with an adenoviral vector containing the MyoD gene (Ad50MyoD; Crucell B.V., Leiden, the Netherlands) using a multiplicity of infection (MOI) of 50–75, cells were forced into myogenesis, according to standardized protocols [15-17]. Myotube cultures were transfected with mixtures of 100 nM of each AON (total AON concentration 100 nM for single and 200 nM for double targeting). Concentration series ranged from 50–250 nM. Polyethylenemine (PEI) was used as transfection reagent to enhance AON transfection *in vitro*, according to the manufacturer's instructions (ExGen 500, Fermentas), with 2.5 μl PEI applied per μg of transfected AON.

### RNA isolation and RT-PCR analysis

RNA was isolated 24–48 hours after transfection using RNA-Bee according to manufacturer's instructions (Campro Scientific). A specific primed RT was performed using the Transcriptor Reverse Transcriptase enzyme (Roche Diagnostics) for 35 minutes at 55°C and 5 minutes at 85°C on ~1 μg of total RNA. PCR analysis was done as described [6]. Skip fragments were isolated from gels and their identity was confirmed with direct sequencing as described [6].

### Dystrophin analysis

Protein samples were isolated 2–8 days after treatment. Non treated control samples were isolated at the same time as the first treated sample. Immunohistochemical and Western blot analyses were performed as previously described [6]. Myosin polyclonal antibody L53 (a gift from Dr. M. van den Hoff, Amsterdam Medical Center, The Netherlands) was used to detect myosin, and MANDYS1 (a gift from Dr. G. Morris, North East Wales Institue, United Kingdom) and NCL-DYS2 (Novacastra Laboratories) were used to detect dystrophin for immuno-histochemical analysis. For Western blot analysis NCL-DYS1 (Novocastra Laboratories) was used to detect dystrophin and MF20 (Developmental Studies Hybridoma Bank (University of Iowa, Iowa City)) to detect myosin. Dystrophin intensities were determined with the Odyssey software (Li-Cor, NE, USA).

## Results

### Exon 45 duplication

**Figure 2:**
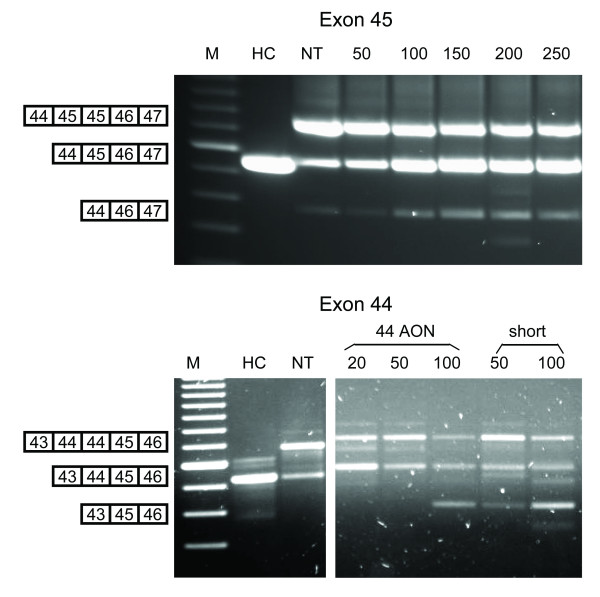
Concentration series of exon 44 and exon 45 AONs. At a concentration of 100 nM (100) and up, levels of wild type transcript increased for the exon 45 duplicated patient (upper panel) compared to the untreated (NT) and 50 nM (50) treated samples. Double exon 45 skipping at low levels was observed for all samples. These levels increase slightly at 100 nM and higher but do not increase further at higher concentrations. For the exon 44 duplicated patient (lower panel), variable low levels of wild type transcripts are present in each sample with the regular exon 44 AON and a short suboptimal AON (short), but as dystrophin was not restored this probably reflects fluctuating levels of the wild type-like artefact. At concentrations that are generally effective (100 nM and up), the levels of original transcript decrease and the amount of double exon 44 skipped transcript increases. M is 100 bp DNA marker, HC is healthy control.

Myotube cultures from a patient with a duplication of exon 45 (Figure 1A) were treated with an exon 45 specific AON. RNA was isolated 2 days after transfection and RT-PCR analysis revealed single exon 45 skipping at significant levels (Figure 1B). Low levels of double exon 45 skipping were observed in both treated and non treated myotubes, which did not increase at higher AON concentrations (Figure 2). Protein samples were collected before, 2 and 5 days after treatment and analyzed by Western blotting using the Dy4 antibody. Clear dystrophin signals were detected in the treated samples (5% and 8% of wild type levels after 2 and 5 days, respectively), whereas no dystrophin was observed in the untreated sample (Figure 1C). The new dystrophin expression was confirmed by immuno-histochemical analysis, where over 80% of treated myotubes was dystrophin-positive (Figure 1D). This is in fact the first time that a wild type dystrophin is generated upon AON treatment. Note that in the non-treated sample low levels of a transcript fragment with similar length of those seen after single exon 45 skipping were observed (Figure 1B, Figure 2 upper panel). This can be explained by PCR artefacts; due to the duplication, the first exon 45 of one PCR strand can anneal to the second exon 45 of the other PCR strand, which would generate a 'wild type-like' product upon amplification. Corresponding to this explanation, we confirmed that no dystrophin protein was detected in the non treated cells by Western blot and immuno-histochemical analyses.

### Exon 44 duplication

The second single exon duplication that we studied for antisense-induced exon skipping involves exon 44 (Figure 3A). In our experience exon 44 skipping is relatively easy, and we have identified various efficient AONs [13]. Myotube cultures from two brothers, both carrying an exon 44 duplication, were treated with these different exon 44 specific AONs. Exon 44 skipping was so efficient that in the vast majority of transcripts both exons 44 were skipped (Figure 3B). In order to enhance single exon 44 skipping we performed a concentration series, used a shorter suboptimal AON (15mer instead of 20mer) and tested different AON:PEI ratios (Figure 2 and data not shown). As for the exon 45 duplication, wild type-like transcripts were observed for each sample at varying levels (Figure 2). Unfortunately, no dystrophin was detected using these lower concentrations, not even for the concentration of 20 nM that appears to induce single exon 44 skipping. This suggests that the different levels of wild type transcripts reflect different levels of the PCR artefact. This is underlined by the finding that at effective concentrations (100 nM and up), the amount of the original duplicated transcript decreases, while the amount of double skipped transcripts increases. These double skipped transcripts generate mainly out-of-frame transcripts (Figure 3A) and no dystrophin was thus introduced (data not shown). There is another strategy to restore the reading frame for these patients. We aimed at inducing the combined skipping of exon 43 in addition to both exons 44 (Figure 3A, third panel). Using a combination of exon 43 and 44 specific AONs, exon 42 was indeed spliced to exon 45 (exon 43-44-44 skip), which leads to an in-frame transcript. Western blot analysis revealed a clear dystrophin signal after treatment with exon 43 and 44 AONs, at 3% of wild type levels (Figure 3C). In addition, dystrophin signals were detected in over 70% of treated myotubes by immuno-histochemical analysis, whereas none of the myotubes stained positive for dystrophin without treatment (Figure 3CD).

### Exon 52–62 duplication

We have previously described a patient who carries an exon 52–62 duplication, located between exons 63 and 64 [18]. As shown in the upper panel of Figure 4, multiexon skipping of the duplicated exons 52–62 would restore the wild type transcript for this patient. Alternatively, the reading frame can be restored to generate a modified, in-frame duplication. This can be achieved either by double exon skipping of the duplicated exons 52 and 62, or by multiexon skipping of the original exons 62, 63 and the duplicated exons 52 and 62 (Figure 4 middle panel). Skipping of the original exon 52 and/or exon 62 or the original exons 52 through 62 will generate out-of-frame transcripts (lower panel Figure 4).

**Figure 5:**
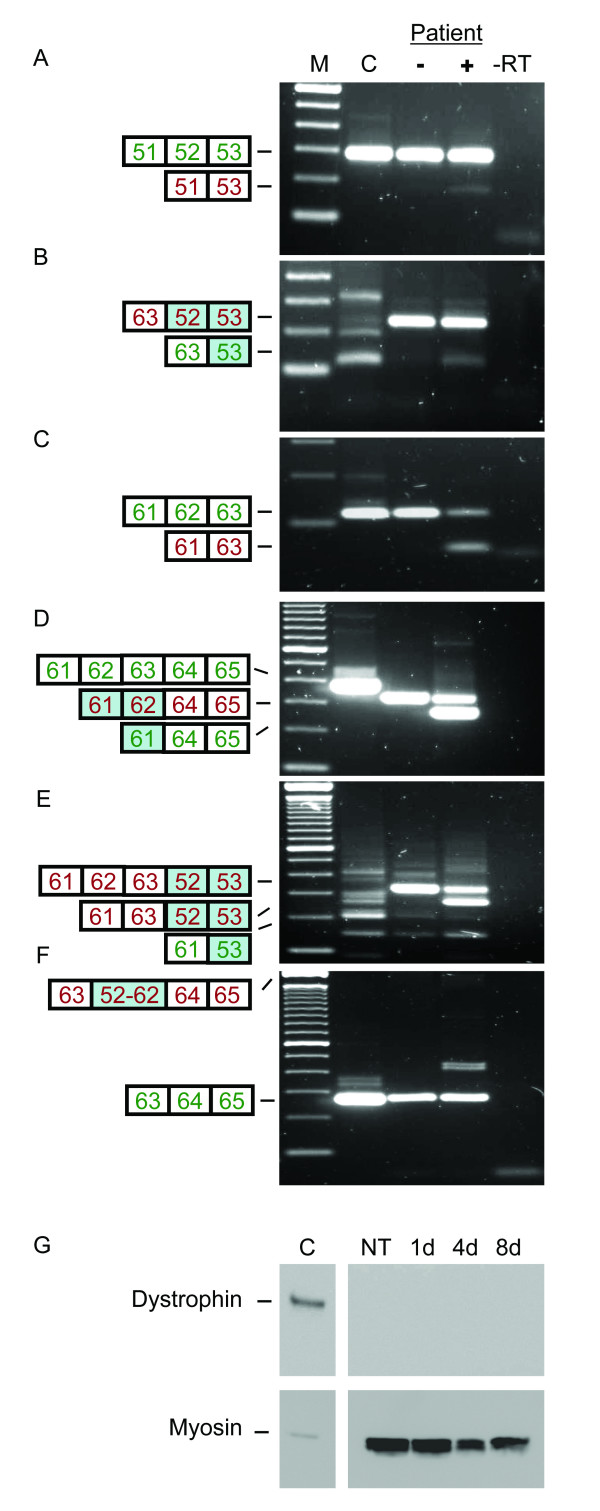
Exon skipping in myotube cultures from a patient with an exon 52–62 duplication. A-F. RT-PCR analysis. After treatment (+) specific skipping of the original and the duplicated exons 52 and 62 was induced in patient myotubes, while these skips were not found before treatment (-) (A-D). Since the duplicated exons are located between exon 63 and 64, the patient appears to have an exon 63 deletion using primers flanking this exon (D). Skipping of the original exons 62, 63, and the duplicated exon 52 could be detected at low levels in untreated myotube cultures (E). After AON treatment, these levels increased and, in addition, single exon 62 skipping was induced. Primers specific for the duplication (B and E) generated only non specific fragments in the control sample, as confirmed by sequencing analysis (C). Using primers flanking the duplication (F) normally spliced transcripts were detected both in untreated and treated patient RNA, albeit at lower levels than the control. The expected fragment of ~1900 bp containing the duplication was not observed (upper marker band is 2 kb). In-frame and out-of-frame transcripts are shown in green and red, respectively. Duplicated exons are shaded in blue. M is 100 bp DNA size marker, -RT is negative control. **G**. Western blot analysis. No dystrophin could be detected in protein isolated from untreated (NT) myotubes, or from myotubes isolated 1, 4 and 8 days after AON treatment. A clear dystrophin signal (dy4) is present in protein isolated from unaffected control myotube cultures (C) (diluted 1:15 to prevent overexposure). Myosin (MF20) signals could be detected in both treated and untreated patient samples, indicating that the differentiation stage of the myotubes was sufficient to allow dystrophin synthesis.

As we have shown previously, it is possible to induce the skipping of multiple exons using AONs targeting the outer exons [7]. Indeed, after treatment of control myotube cultures with AONs specific for exon 52 and exon 62, skipping of the stretch of exons between exons 52 and 62 was observed occasionally (twice in 18 samples), showing that this multiexon skipping is, at least in principle, feasible (data not shown). This multiexon skipping was not detected is untreated control samples (n = 10) nor were intermediate products, arising from the skipping of exons within the targeted region, observed in treated or untreated samples. We then treated myotube cultures of the patient with the exon 52 and exon 62 AONs and RNA was analyzed by RT-PCR using primer pairs flanking the duplication breakpoints and the original exons (Figure 5A–F). For both exon 52 and exon 62, skipping of the original as well as the duplicated exons was observed (Figure 5A–E). However, the reading frame is restored only by the combined skipping of the duplicated exons 52 and 62 in a transcript where the original exons 52 and 62 are maintained. To verify this, one would have to analyze a stretch of exons including the original exons 51 to 63, the duplicated exons 52 to 62 and the original exon 64, which is a fragment of over 4 kb in length. This was attempted without success, so we aimed at amplification across the duplication in order to see a decrease in duplicated transcripts after AON treatment (Figure 5F). Unexpectedly, equal levels of correctly spliced exon 63 were observed in both untreated and treated patient myotubes. However, these levels were lower than those observed for the control myotube cultures. Unfortunately, we did not observe the ~1900 bp fragment containing the duplication in treated or non treated RNA from the patient. On the basis of our RT-PCR data we could thus not conclude whether the wild type transcript or the reading frame was restored. Therefore, Western blot analysis was used to detect restoration of the dystrophin synthesis (Figure 5G). No dystrophin was detected for up to 8 days after treatment, or in untreated myotubes. Antibodies against myosin were used to confirm that the differentiation of the myotubes was sufficient to allow dystrophin synthesis.

## Discussion

In principle, duplications are ideal targets for the exon skipping therapy, as normal transcripts and wild type dystrophin are generated after AON treatment. In addition, we hypothesized that for single exon duplications, exon skipping might be more efficient per se, as skipping of either one of the duplicated exons will restore the transcript. This indeed turned out to be feasible for an exon 45 duplication patient. Thus, in this case we achieved the ideal therapeutic result: patient myotubes producing wild type dystrophin after AON treatment. On the other hand, for two exon 44 duplication patients, exon 44 skipping turned out to be so efficient that mainly transcripts were generated in which both duplicated exons were skipped. Under suboptimal transfection conditions we observed no skip at all. We note that exon 44 skipping has consistently been very efficient in both patient and control cell lines [6,13] and unpublished results], which might explain why under optimal conditions both exons were skipped in these exon 44 duplicated cells. This phenomenon has never been observed for the exon 45 duplication. Here, we mainly observed single exon 45 skipping after AON treatment, regardless of whether the AON induced very efficient (> 80% skipping levels as determined with the Agilent lab on a chip system) or less efficient exon 45 skipping (~10% skipping levels) in control myotube cultures. This suggests that the occurrence of single or double exon skipping in single exon duplications is dependent on the duplicated exon itself. Splicing of individual exons is influenced by various exonic and intronic splicing enhancers and silencers. Some exons are more dependent of such regulatory elements than others for proper inclusion in the mRNA, explaining why different duplicated exons respond differently to AON treatment. Finally, skipping efficiency may even be different for unrelated patients carrying the 'same' single exon duplication as the intronic breakpoints (and thus the presence or absence of intronic enhancers) may vary.

Multiple exon duplications are more challenging, as the normal transcript can only be restored by multiexon skipping, which is less efficient than single exon skipping and not feasible for each and every stretch of exons [12]. Alternatively, the reading frame of the duplication can be restored by targeting a smaller amount of duplicated exons. The difficulty is that in addition to the targeted exon (the skipping of which will restore the open reading frame) there is another similar exon, for which the skipping is frame-disrupting. It may be hard to identify which of these exons are skipped. In addition, the analysis may be obscured due to PCR artefacts. As both the original and the duplicated part of the transcript are similar to each other, they can anneal and give rise to products that are similar to 'wild type' fragments. It should be noted that we observed these seemingly normal fragments in non treated RNA samples for each patient. For the single exon duplication, there are no exon junctions in the restored transcript that are not also present in the duplicated transcript. Thus it is impossible to perform a PCR specific for the skipped RNAs and so discriminate between artefacts and skipped fragments. However, in line with the severity of the disease, no dystrophin was detected in untreated samples. Due to the nature of the duplication (exon 52–62 located between exon 63 and exon 64), we could discriminate between artefacts and restored fragments using primers in exon 63. Surprisingly, wild type fragments were observed both in treated and untreated samples, albeit at low levels. As not a hint of dystrophin could be observed on the Western blot, these levels are not sufficient to generate detectable amounts of protein. Thus, novel expression of the dystrophin protein can here be used as outcome measurement to assess whether the reading frame is restored for multiple exon duplications. The resulting dystrophin would likely be functional, as BMD patients with in-frame duplications have been reported. However, it cannot be excluded that the additional domains encoded by the duplicated exons may disrupt the secondary structure of the protein as there are DMD patients carrying in-frame duplications as well [5].

The multiexon duplication patient in this study was extra challenging, since the duplicated exons 52–62 are not located between exons 62 and 63, but between exons 63 and 64 [18]. Therefore, even though we were unable to restore dystrophin for this particular patient, other multiexon duplications may be easier to correct, especially those involving only a limited number of exons.

## Conclusion

Exon skipping for patients with small duplications offers an ideally therapeutic approach, as it allows the restoration of the wild type transcript. Indeed we here achieved this by skipping a single copy of exon 45. If the skipping is too rigorous, multiexon skipping including adjacent exons can alternatively be applied to restore the open reading frame, achieving a BMD-like restoration product similar to those obtained after the correction of out-of-frame deletions. For larger multi-exon duplications restoration of the wild type transcript and/or the reading frame is more challenging and its achievement will depend on the nature and the number of exons duplicated.

## Abbreviations

AON Antisense oligonucleotide

BMD Becker muscular dystrophy

DMD Duchenne muscular dystrophy

MOI Multiplicity of infection

PEI Polyethylenimine

RT-PCR Reverse transcriptase polymerase chain reaction

## Competing interests

The author(s) declare that they have no competing interests.

## Authors' contributions

AA was involved in the double and multiexon skipping studies, participated in the design and coordination of the study and drafted the manuscript. AJ carried out the single exon skipping studies. GJvO supervised the group and helped to draft the manuscript. JvD was involved in the design and coordination of the study and helped to draft the manuscript.

## Pre-publication history

The pre-publication history for this paper can be accessed here:


